# Supplementary figures and images for: Influence of Lab Adapted Natural Diet and Microbiota on Life History and Metabolic Phenotype of Drosophila melanogaster
Source: Microorganisms. 2020 Dec 11;8(12):1972. doi: 10.3390/microorganisms8121972 (PMC7763083; doi:10.3390/microorganisms8121972)

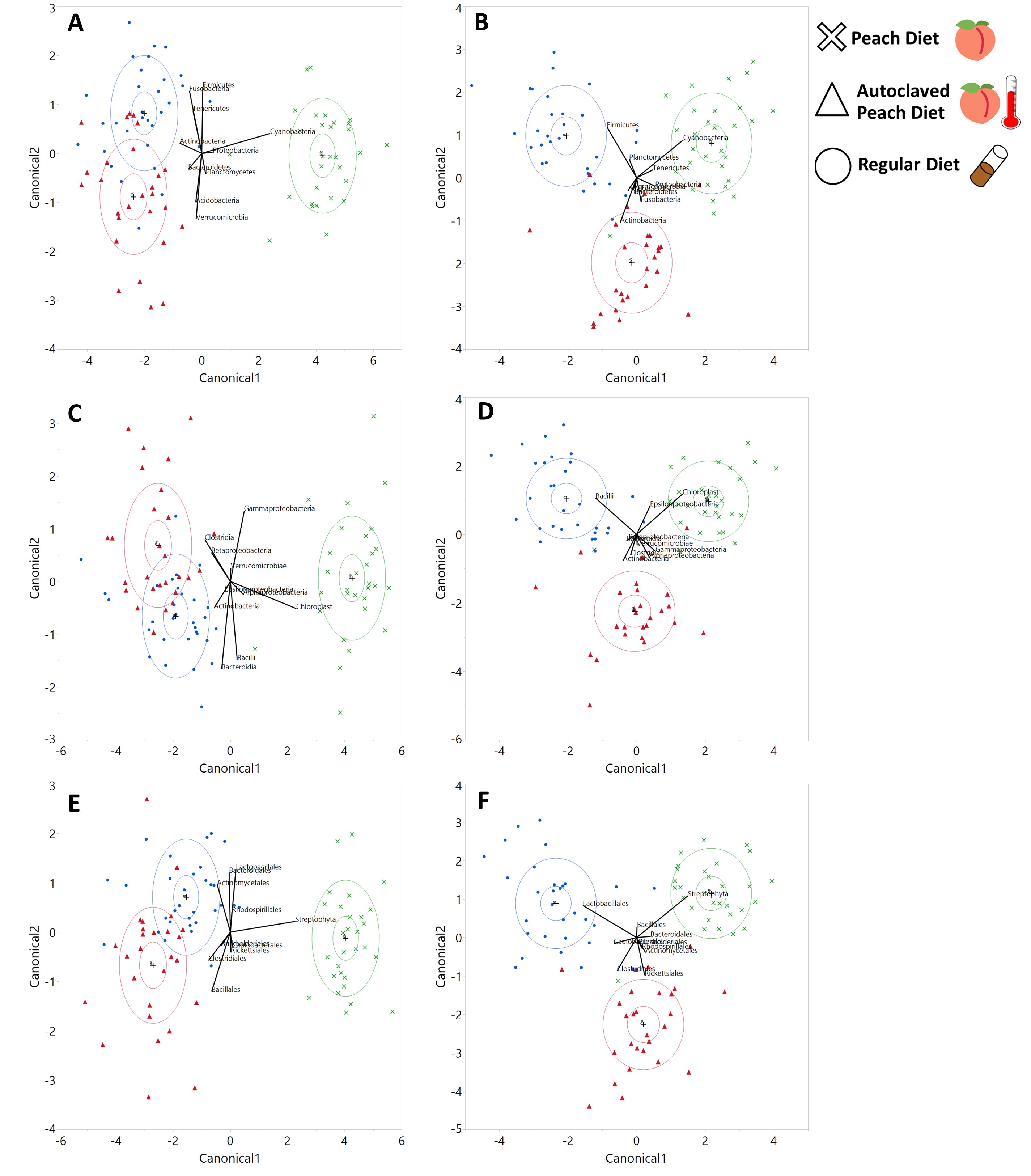

Supplement: Supplementary file 1 [file microorganisms-08-01972-s001.zip › figure supplement 1.jpg]

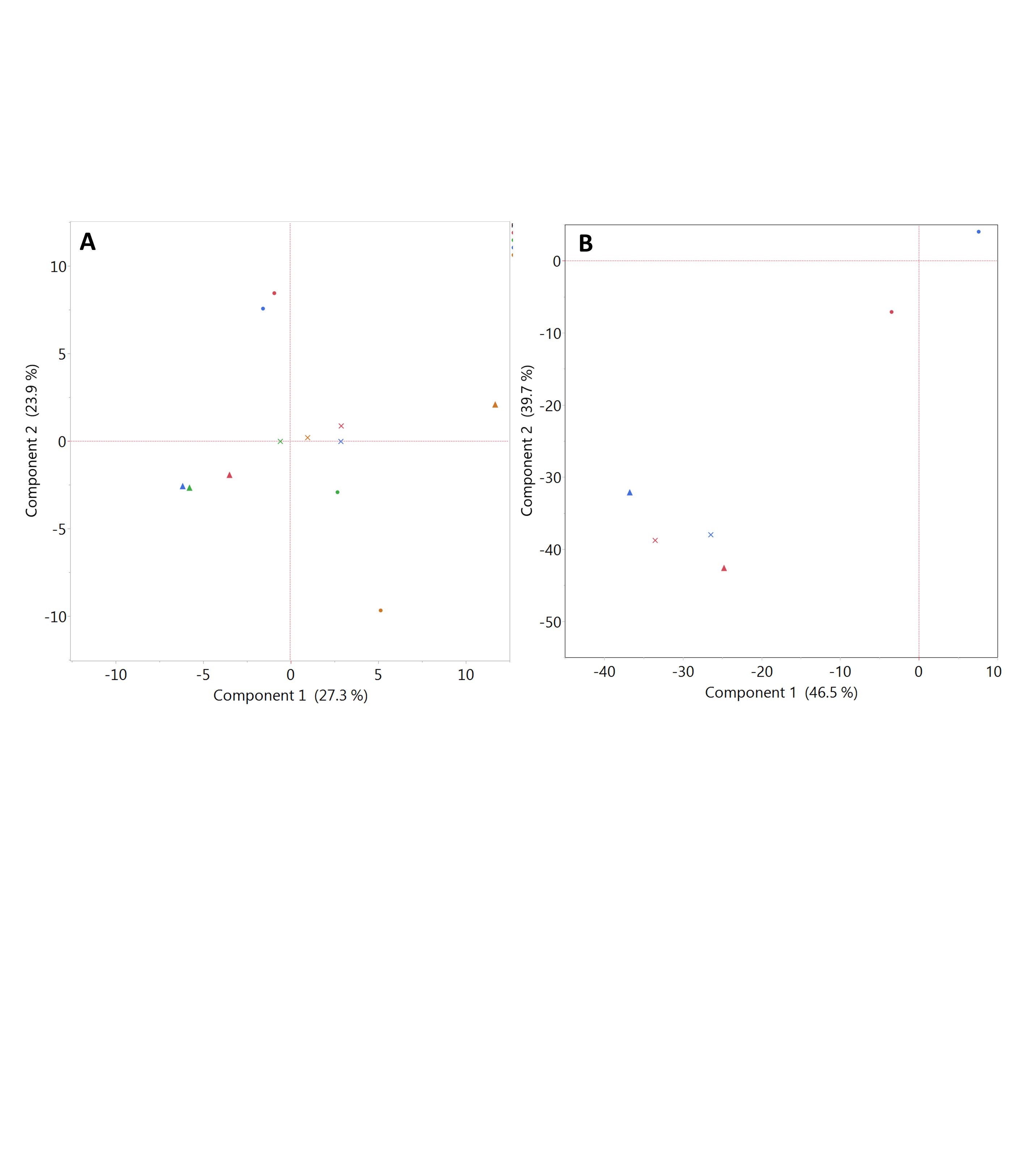

Supplement: Supplementary file 1 [file microorganisms-08-01972-s001.zip › figure supplement 10.jpg]

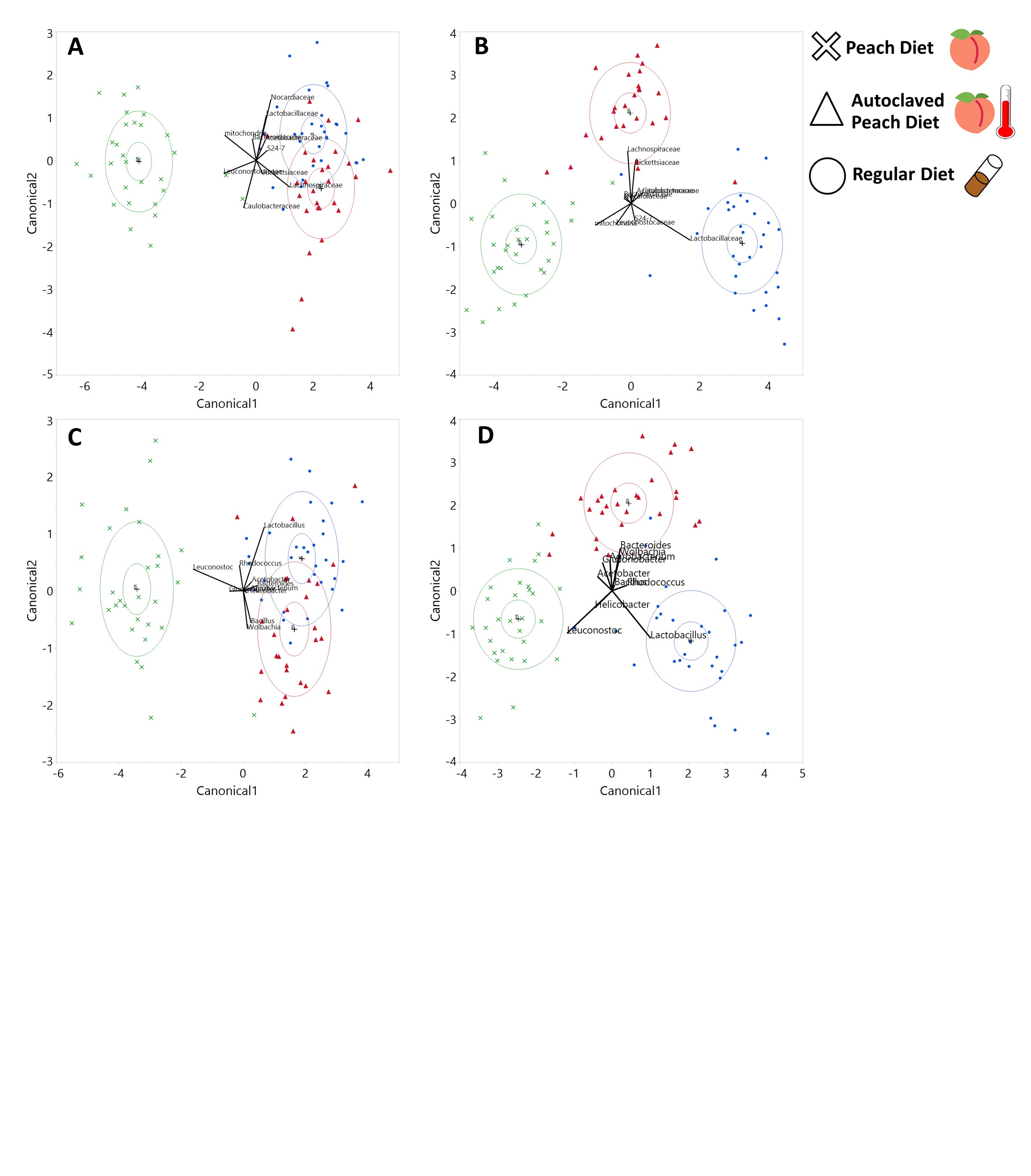

Supplement: Supplementary file 1 [file microorganisms-08-01972-s001.zip › figure supplement 2.jpg]

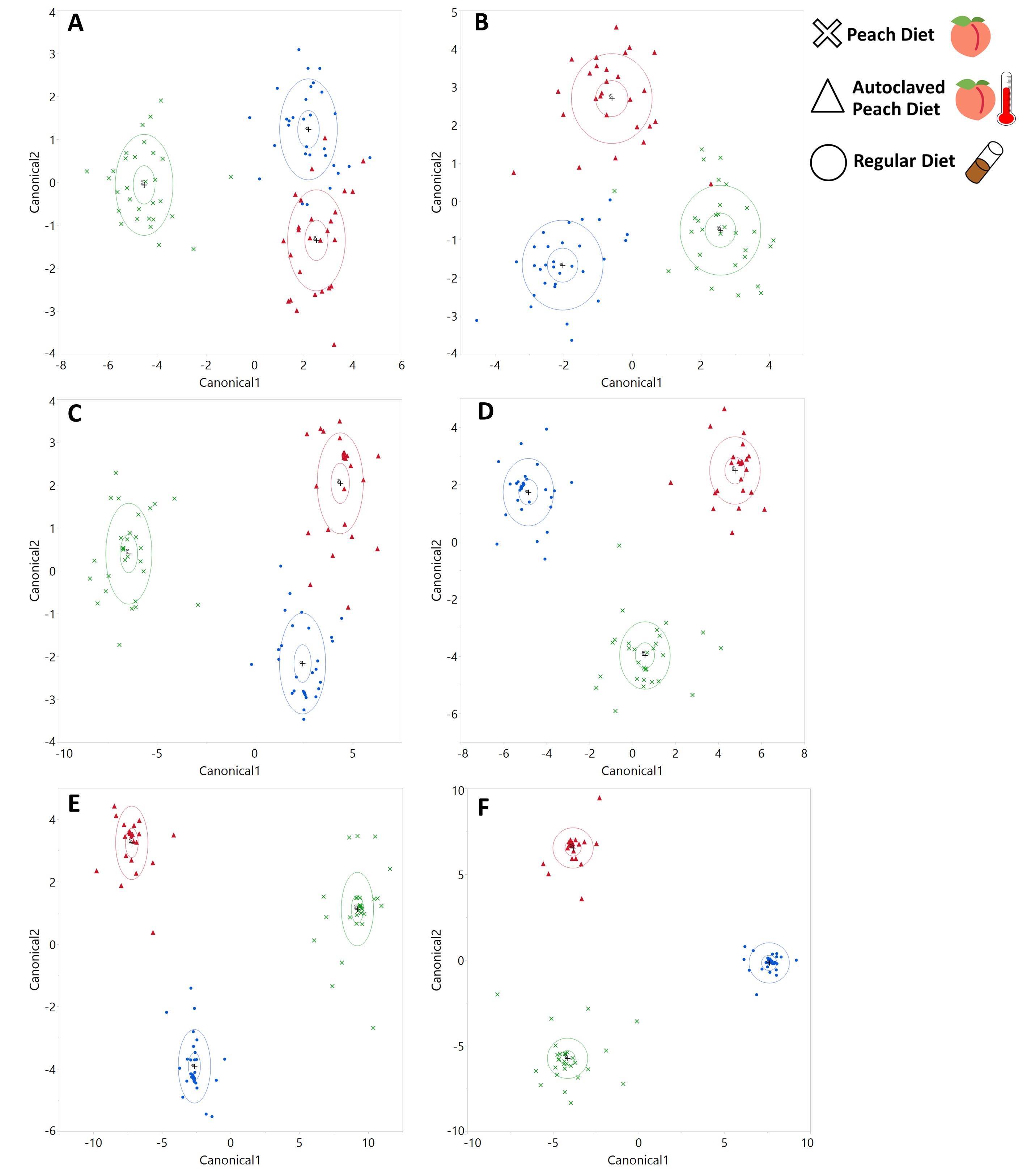

Supplement: Supplementary file 1 [file microorganisms-08-01972-s001.zip › figure supplement 3.jpg]

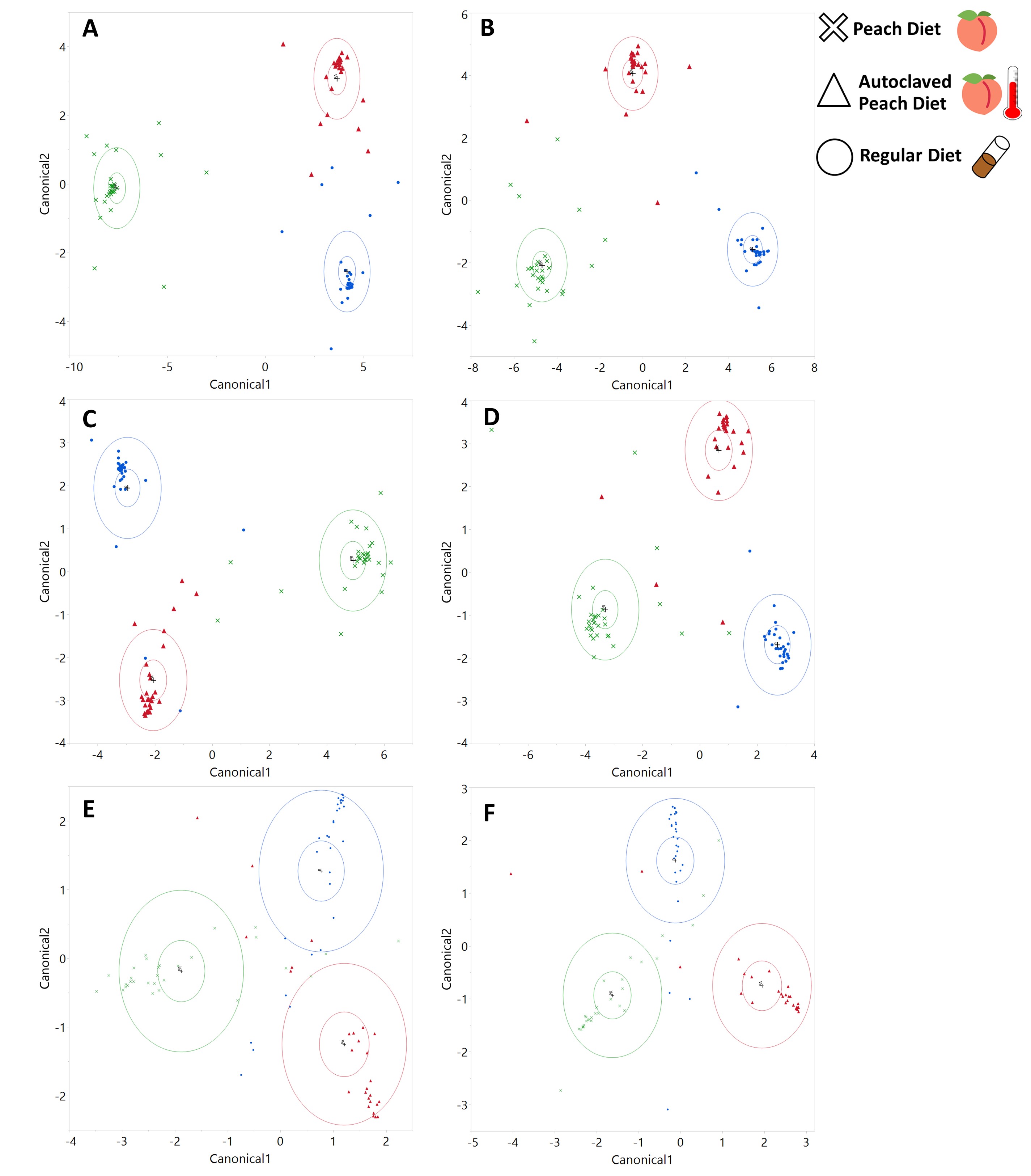

Supplement: Supplementary file 1 [file microorganisms-08-01972-s001.zip › figure supplement 4.jpg]

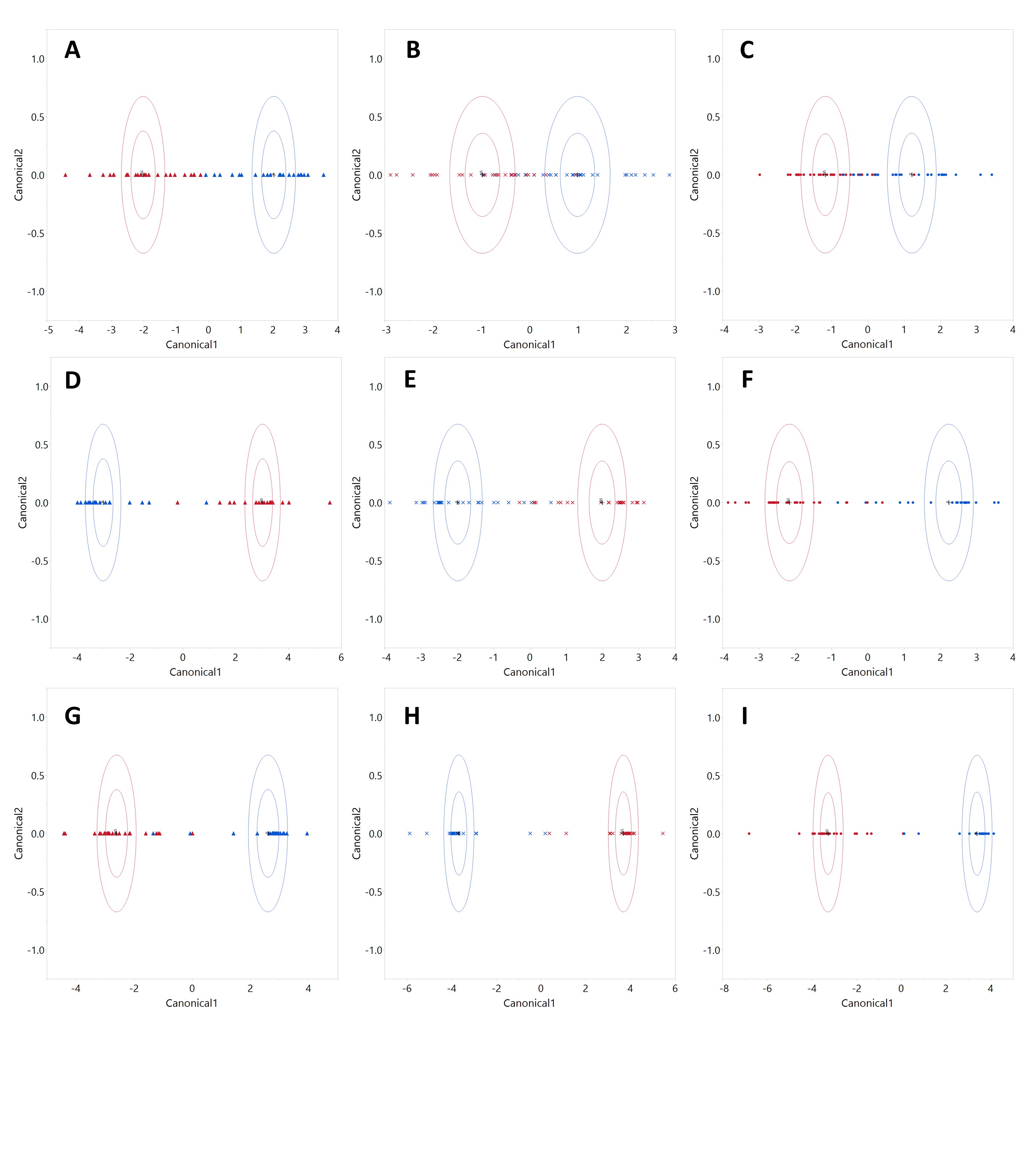

Supplement: Supplementary file 1 [file microorganisms-08-01972-s001.zip › figure supplement 5.jpg]

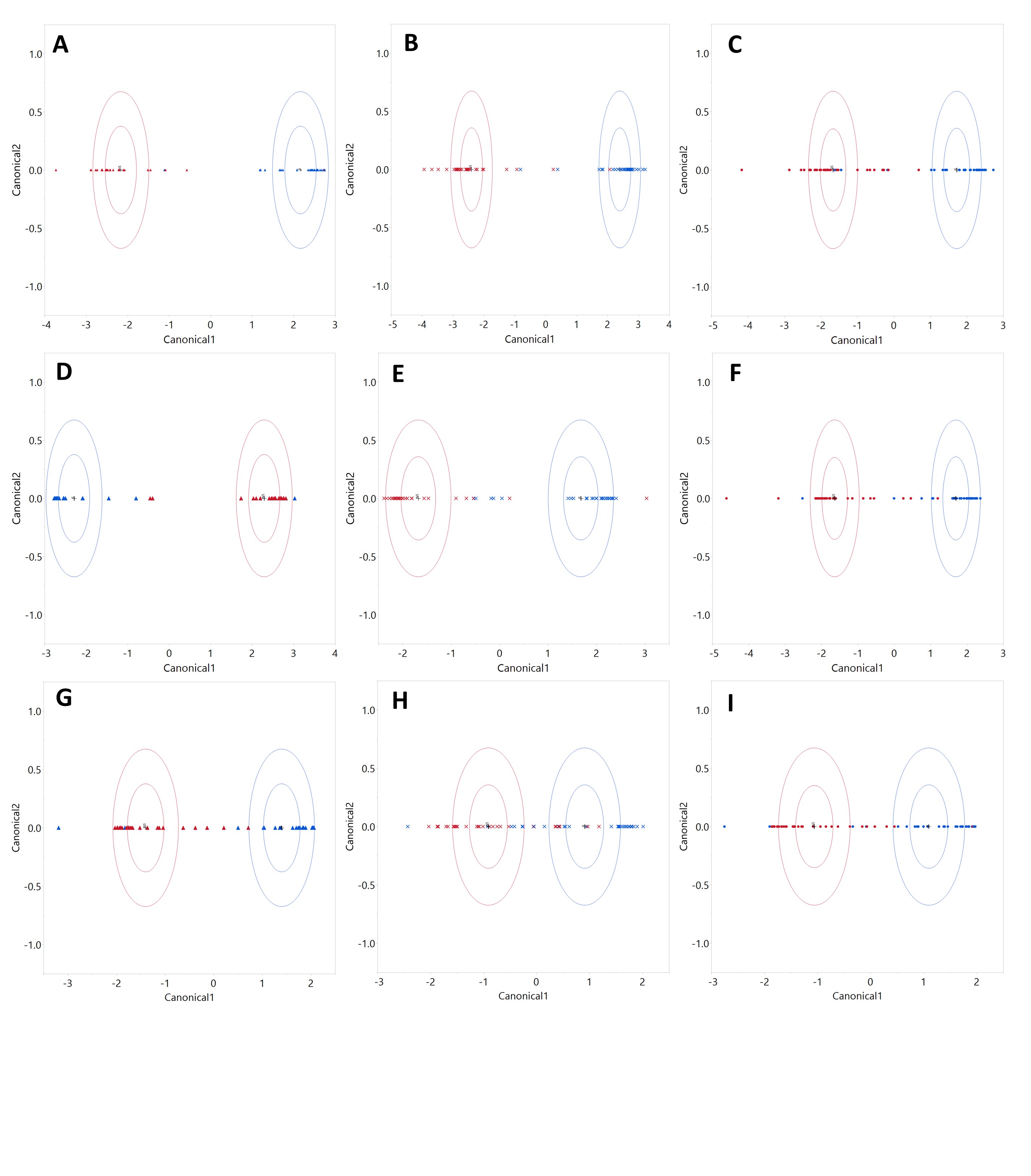

Supplement: Supplementary file 1 [file microorganisms-08-01972-s001.zip › figure supplement 6.jpg]

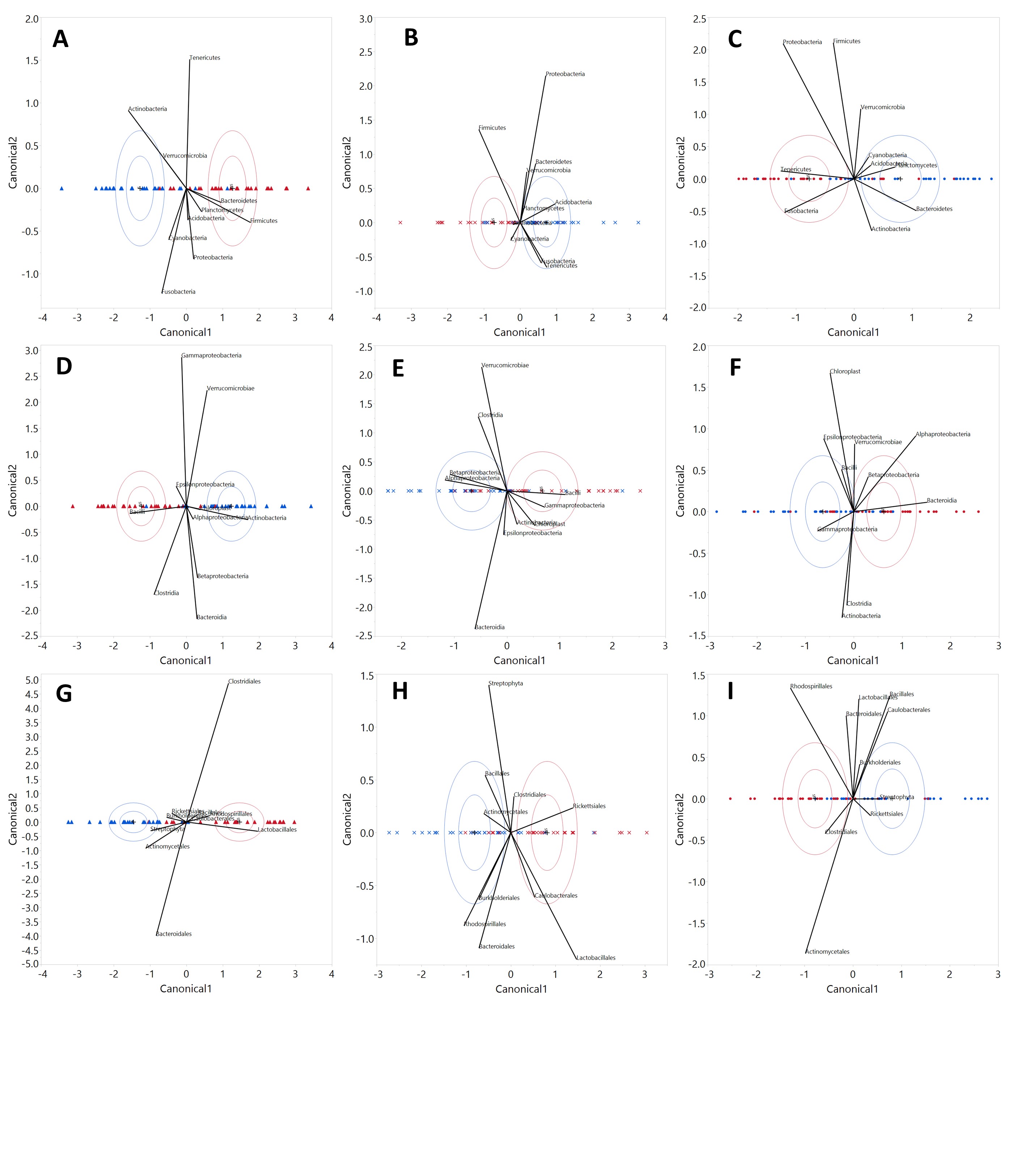

Supplement: Supplementary file 1 [file microorganisms-08-01972-s001.zip › figure supplement 7.jpg]

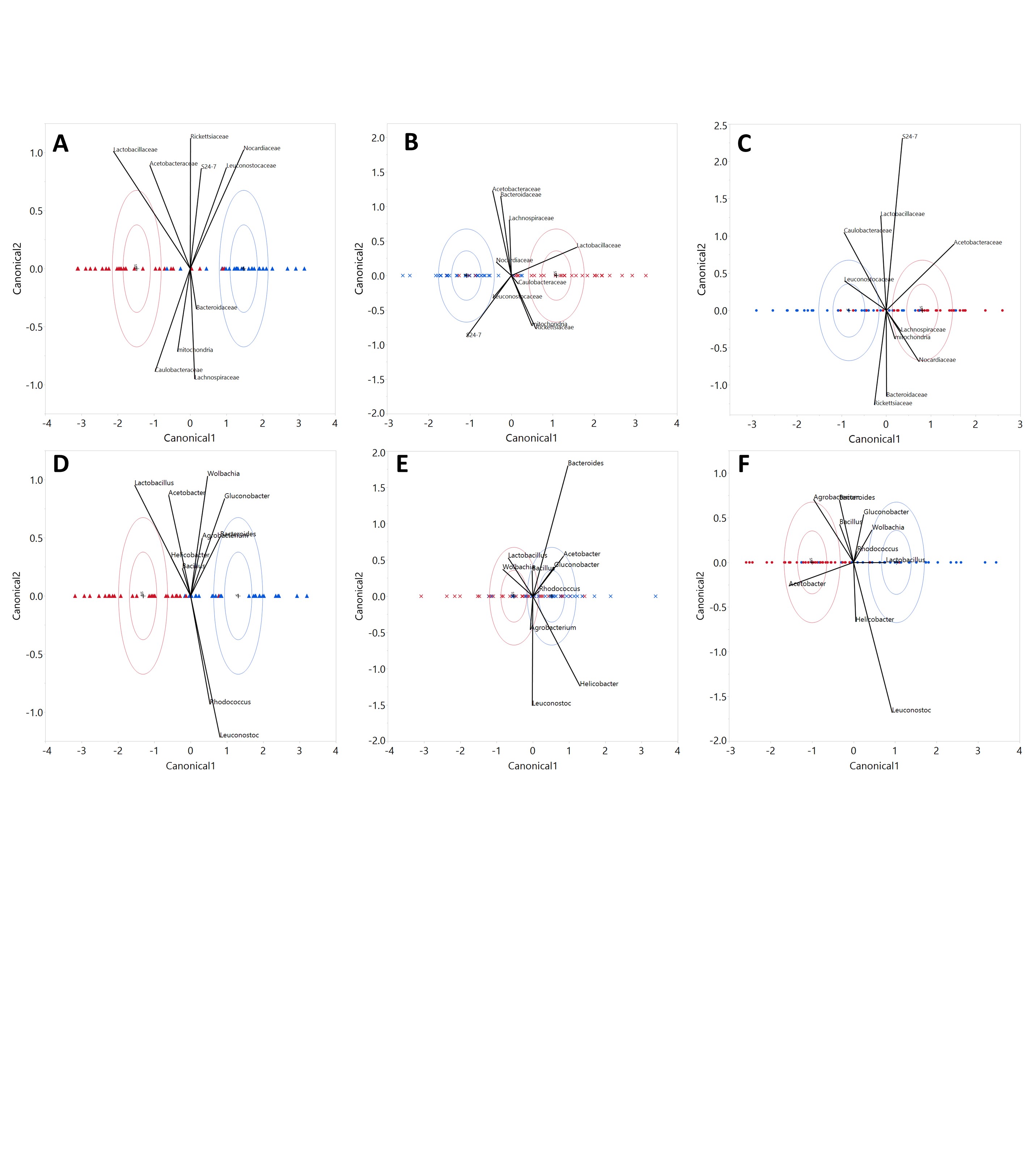

Supplement: Supplementary file 1 [file microorganisms-08-01972-s001.zip › figure supplement 8.jpg]

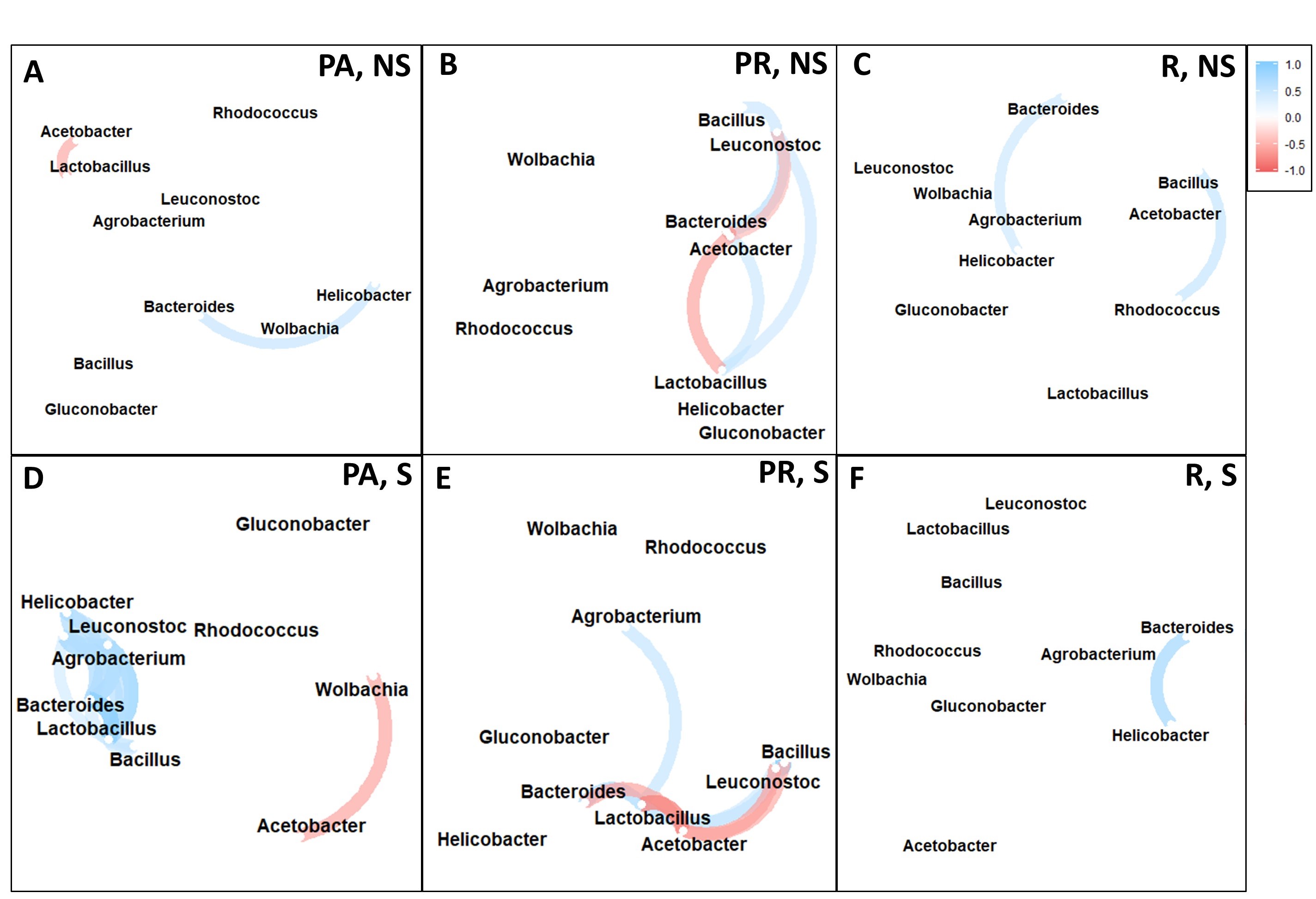

Supplement: Supplementary file 1 [file microorganisms-08-01972-s001.zip › figure supplement 9.jpg]
